# From Sugar Spikes to Pressure Peaks: Navigating the World of Diabetes, Hypertension, Obesity, and Kidney Health

**DOI:** 10.7759/cureus.57241

**Published:** 2024-03-30

**Authors:** Nay Phone Hlyan, Talha Arif, Saina S Jaufar, Abdur Rehman, Biruk D Ayalew, Biniyam J Batu, Muhidin I Hundesa, May Su Hlaing, Hamza Islam, Rabia Islam, Abdullah Shehryar, Maria Quinn

**Affiliations:** 1 General Surgery, Barts Health National Health Service (NHS) Trust, London, GBR; 2 Accident and Emergency, Imran Idrees Teaching Hospital, Sialkot, PAK; 3 Family Medicine and General Surgery, Vitebsk State Medical University, Vitebsk, BLR; 4 Surgery, Mayo Hospital, Lahore, PAK; 5 Internal Medicine, St. Paul's Hospital Millennium Medical College, Addis Ababa, ETH; 6 General Practice, St. Paul's Hospital Millennium Medical College, Addis Ababa, ETH; 7 General Practice, Ministry of Health, Addis Ababa, ETH; 8 Geriatrics, United Lincolnshire Hospitals National Health Service (NHS) Trust, Boston, GBR; 9 Internal Medicine, Punjab Medical College, Faisalabad, PAK; 10 Research, Faisalabad Medical University, Faisalabad, PAK; 11 Internal Medicine, Allama Iqbal Medical College, Lahore, PAK; 12 Internal Medicine, Jinnah Hospital, Lahore, PAK

**Keywords:** multidisciplinary approach, integrated care, pharmacogenomics, personalized medicine, chronic kidney disease, obesity, hypertension, diabetes

## Abstract

Diabetes, hypertension, obesity, and chronic kidney disease (CKD) are major public health challenges globally, contributing significantly to morbidity and mortality. The co-occurrence and interplay among these conditions exacerbate health outcomes, highlighting the need for an integrated understanding and approach to management. This narrative review aims to explore the complex relationships between diabetes, hypertension, obesity, and CKD, elucidating their collective impact on health. It discusses the epidemiological trends, underlying pathophysiological mechanisms, genetic predispositions, current treatment strategies, and the future direction of research and therapy. An extensive review of current literature was conducted, focusing on the epidemiology, pathophysiology, risk factors, diagnosis, and treatment of diabetes, hypertension, obesity, and CKD. Additionally, the review delves into the genetic and molecular biology underlying these conditions, the potential for personalized medicine, and the importance of a multidisciplinary approach to care.

The review identifies key areas where these conditions intersect, enhancing disease progression and complicating management. It highlights the role of genetic and environmental factors in disease etiology, the critical need for personalized treatment strategies, and the gaps in current management approaches. Innovations in pharmacotherapy, monitoring technologies, and the potential of pharmacogenomics are discussed as avenues for advancing patient care. Diabetes, hypertension, obesity, and CKD are intricately linked, necessitating an integrated, patient-centered approach to care that goes beyond traditional treatment modalities. Future research should focus on collaborative models and interdisciplinary strategies to address the multifaceted challenges posed by these conditions. Emphasizing personalized medicine and leveraging technological advancements offer promising pathways to improve outcomes and reduce the global health burden of these metabolic disorders.

## Introduction and background

Diabetes mellitus, hypertension, obesity, and chronic kidney disease (CKD) are major public health concerns worldwide. Diabetes is characterized by chronic hyperglycemia resulting from defects in insulin secretion, insulin action, or both [1]. The global prevalence of diabetes has risen dramatically from 4.7% in 1980 to 8.5% in 2014 [2]. Hypertension, defined as systolic blood pressure ≥140 mm Hg or diastolic blood pressure ≥90 mm Hg, affected 1.13 billion people globally in 2015 [3]. The prevalence of obesity has nearly tripled since 1975, with over 650 million adults being obese in 2016 [4]. CKD affected 9.1% of the global population in 2017 [5]. Together, these conditions account for substantial morbidity and mortality.

These conditions often co-occur and interact to worsen health outcomes. Obesity and diabetes are closely linked; excess body weight is responsible for over 60% of cases of type 2 diabetes [6]. Diabetes and hypertension influence each other; diabetes promotes the development of hypertension while hypertension exacerbates diabetic microvascular complications [7]. Hypertension and obesity both increase the strain on the kidneys and the risk of CKD [5]. Diabetic kidney disease occurs in 20-40% of people with diabetes [8]. The interconnected nature of these conditions creates a snowball effect, where one condition promotes others and their collective burden accelerates adverse outcomes. 

The complex relationships between these highly prevalent conditions mean their collective impact is difficult to fully convey. This narrative review aims to map out these relationships by discussing the underlying links between diabetes, hypertension, obesity, and CKD. We detail how they influence one another, highlighting key mechanisms and epidemiological data. We also explore how their interconnected nature relates to clinical outcomes like cardiovascular disease, renal failure, and mortality. Finally, we suggest ways to leverage these connections for more effective screening, prevention, diagnosis, and management. Examining these conditions together provides insights not apparent when they are studied in isolation. It enables a better understanding of real-world scenarios faced in clinical practice involving patients with overlapping diabetes, hypertension, obesity, and kidney disease.

## Review

Diabetes: managing sugar spikes

Diabetes mellitus refers to metabolic disorders characterized by chronic high blood glucose levels (hyperglycemia) resulting from defects in insulin secretion, insulin action, or both [9]. The two main types are type 1 diabetes mellitus (T1DM) and type 2 diabetes mellitus (T2DM). Gestational diabetes mellitus (GDM) occurs during pregnancy [10].

Types of Diabetes: Type 1, Type 2, and Gestational

T1DM accounts for 5-10% of cases and results from the autoimmune destruction of pancreatic beta cells, which causes insulin deficiency. T2DM is more prevalent and accounts for 90-95% of cases. It results from insulin resistance coupled with inadequate compensatory insulin secretion. GDM occurs in 3-5% of pregnancies due to hormonal changes that antagonize insulin action [11].

Pathophysiology and Risk Factors 

Risk factors for T1DM include genetics and environmental triggers. Risk factors for T2DM include family history, obesity, physical inactivity, older age, and ethnicity. GDM shares risk factors with T2DM and has additional risk from prior GDM or having a macrosomic baby [12].

 Diagnosis and Monitoring

Diabetes screening relies on tests to measure plasma glucose levels, such as fasting plasma glucose, 2‐hour plasma glucose during an oral glucose tolerance test, or HbA1c. HbA1c reflects average glycemia over ~3 months. Diagnostic cutoffs are fasting glucose ≥126 mg/dL, 2‐hour glucose ≥200 mg/dL, or HbA1c ≥6.5% [9].

Treatment Strategies: Medication, Diet, and Lifestyle

**Figure 1:**
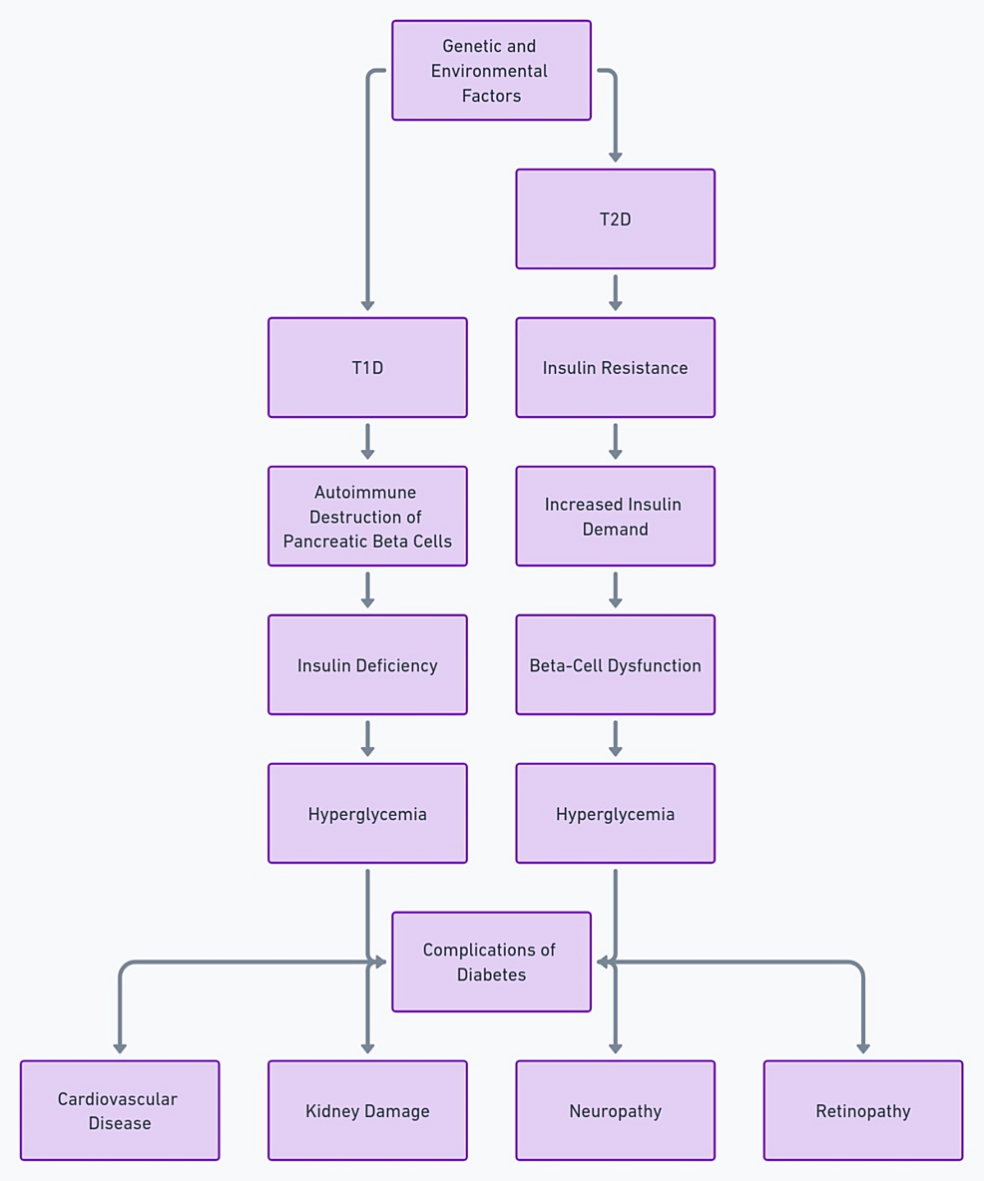
This flowchart delineates the distinct pathways through which genetic and environmental factors precipitate the onset of type 1 and type 2 diabetes, leading to various complications, by illustrating the sequence of events from autoimmune destruction or insulin resistance to hyperglycemia and subsequent health issues. This image is produced by the authors.

Treatment for T1DM relies on exogenous insulin administration. For T2DM it starts with lifestyle modifications (weight loss, exercise, healthy diet) along with metformin. Other oral medications, injectable therapies like GLP-1 agonists, or insulin are added as needed to achieve glycemic targets [13]. A summary is given in Figure 1.

Hypertension: understanding pressure peaks 

Hypertension, commonly defined as sustained elevated blood pressure ≥130/80 mmHg, is highly prevalent affecting ~1 billion adults globally [14]. It is a major modifiable risk factor for stroke, myocardial infarction, heart failure, and CKD.

Epidemiology and Risk Factors of Hypertension

The prevalence of hypertension rises with age, affecting more than 60% of people over 60 years old. Key risk factors include excess body weight, excess dietary sodium intake, reduced physical activity, genetics, and social determinants of health [15]. 

Pathophysiology of High Blood Pressure

In 95% of cases, the underlying cause is unknown (primary hypertension) likely stemming from complex interactions between environmental and genetic factors influencing renal salt handling and vascular function. Secondary hypertension accounts for 5% of cases resulting from discrete causes like renal parenchymal disease, renovascular disease, aldosteronism, phaeochromocytoma, obstructive sleep apnea, or thyroid disorders [14].

Diagnostic Criteria and Monitoring 

Accurate blood pressure measurement is key for diagnosis. Ambulatory blood pressure monitoring can refine risk stratification. Basic laboratory tests help screen for secondary causes. Monitoring response to antihypertensive medication is also important [14]. 

Management: Pharmacological and Non-Pharmacological Approaches

Lifestyle changes, including weight reduction, dietary sodium restriction, increased fruit/vegetable intake, moderation of alcohol intake, and increased physical activity, are recommended for all patients with hypertension. If glycemic targets are not met within 3 months of lifestyle changes, antihypertensive medications are initiated, typically starting with thiazide diuretics, angiotensin-converting enzyme (ACE) inhibitors, angiotensin receptor blockers, or calcium channel blockers [16].

Obesity: a growing global challenge

Overweight and obesity, defined by abnormal or excessive body fat accumulation posing health risks, have reached epidemic proportions globally. In 2016, 39% of adults aged 18 years and older were overweight and 13% were obese [4]. 

Defining and Measuring Obesity

Obesity is commonly assessed using body mass index (BMI kg/m2) cutoffs of ≥25 kg/m2 for overweight and ≥30 kg/m2 for obesity. However, measures like waist circumference and waist-hip ratio better capture abdominal/visceral obesity, which is more strongly tied to metabolic abnormalities [17].

Causes and Consequences of Obesity

Fundamentally, obesity results from chronic caloric intake exceeding energy expenditure. Myriad genetic, lifestyle and environmental factors drive this imbalance, producing adverse health consequences like T2DM, cardiovascular disease, cancers, mental illness, and reduced life expectancy [18].

Obesity Management: Lifestyle, Medical, and Surgical Interventions

Lifestyle interventions targeting diet, physical activity, and behavior change have modest efficacy for weight loss and maintenance. Pharmacotherapy can augment weight loss. Bariatric surgery leads to substantial weight reduction for severe obesity where lifestyle interventions have failed [19].

Prevention Strategies and Public Health Implications 

Population-level policy interventions targeting food environments, built environments, and socioeconomic factors enable healthier lifestyles to curb rising obesity rates. Such upstream approaches, along with personalized solutions and obesity education campaigns, are key [4]. 

Kidney health: the renal realities

CKD is increasingly common affecting ~10% of people globally. Diabetes and hypertension are major risk factors for CKD. Early detection and management of CKD, along with treatment of underlying drivers, can slow disease progression [5].

CKD and Its Stages 

CKD involves progressive structural and functional kidney damage resulting in albuminuria, decreased glomerular filtration rate (GFR), and other complications over months to years. CKD stages range from mild renal abnormalities (stage 1) to total kidney failure necessitating dialysis or transplantation (stage 5) [20]. 

The Impact of Diabetes and Hypertension on Kidney Function

Diabetes and hypertension foster CKD progression through mechanisms like elevated intraglomerular pressure and activation of pathways driving glomerulosclerosis and tubulointerstitial fibrosis. Good control of blood glucose and blood pressure can delay CKD in these high-risk groups [8].

Diagnosis and Monitoring of Kidney Health 

Screening for CKD involves estimating GFR from serum creatinine-based equations and testing for albuminuria. Repeat testing determines progression. Imaging and kidney biopsies clarify causes as needed [20].

Treatment Approaches and Renal Replacement Therapies 

ACE inhibitors and angiotensin receptor blockers help slow CKD progression by targeting pathways that drive kidney damage, especially in proteinuric cases. As kidney function declines further, renal replacement therapy with dialysis or transplantation is ultimately needed [20].

Interplay of metabolic disorders

Diabetes, hypertension, obesity, and CKD interact to exacerbate the progression of each other. Metabolic syndrome describes the clustering of interconnected risk factors driving adverse cardiovascular and renal outcomes [21]. 

Relationship Between Diabetes, Hypertension, Obesity, and CKD

Obesity, diabetes, and hypertension are dominant risks for new onset CKD and its progression. Mechanisms like inflammation, oxidative stress, and lipotoxicity underlie their synergistic impact. Kidney disease further elevates cardiovascular risk in these groups via disordered mineral metabolism, hypertension, and vascular calcification [8].

Metabolic Syndrome and Its Implications

Metabolic syndrome comprises central obesity, hyperglycemia, hypertension, and dyslipidemia. It affects ~20-25% of the global adult population conferring a 2-fold increase in cardiovascular outcomes and a 1.5-fold increase in all-cause mortality [3].

Strategies for Integrated Care and Management

Complex care needs demand integrated, multidisciplinary care models spanning primary and specialty care to concurrently address risks like glycemia and blood pressure while providing patient education and psychosocial support [22].

Genetics and molecular biology

Genetic Predispositions and Risk Factors

Diabetes, obesity, hypertension, and CKD exhibit significant heritability, indicating that genetic predispositions play a critical role in the risk and development of these conditions. Genome-wide association studies (GWAS) have identified numerous genetic loci associated with T2DM, obesity, and hypertension, highlighting the polygenic nature of these disorders. For instance, variants in the TCF7L2 gene have been shown to significantly increase the risk of developing T2DM [23]. Similarly, the FTO gene has been linked to obesity susceptibility, affecting energy homeostasis and metabolism [24]. In hypertension, variants in genes related to salt sensitivity and renin-angiotensin-aldosterone system (RAAS) function have been implicated [25]. These genetic insights underscore the importance of understanding individual genetic profiles in assessing disease risk and progression.

Molecular Mechanisms Underlying Disease Progression

The molecular pathways involved in the progression of metabolic disorders are complex and multifaceted. In diabetes, the destruction of pancreatic beta cells in T1DM and insulin resistance in T2DM involve inflammatory pathways, oxidative stress, and lipotoxicity, leading to beta-cell dysfunction and impaired glucose uptake [26]. Obesity contributes to metabolic syndrome and T2DM through mechanisms such as adipokine imbalance, inflammation, and altered lipid metabolism, which exacerbate insulin resistance [27]. The molecular basis of hypertension involves dysregulation of vascular tone, endothelial dysfunction, and overactivity of the sympathetic nervous system and RAAS [28]. Understanding these pathways is crucial for the development of targeted therapies and interventions.

Personalized Medicine and Pharmacogenomics

**Figure 2:**
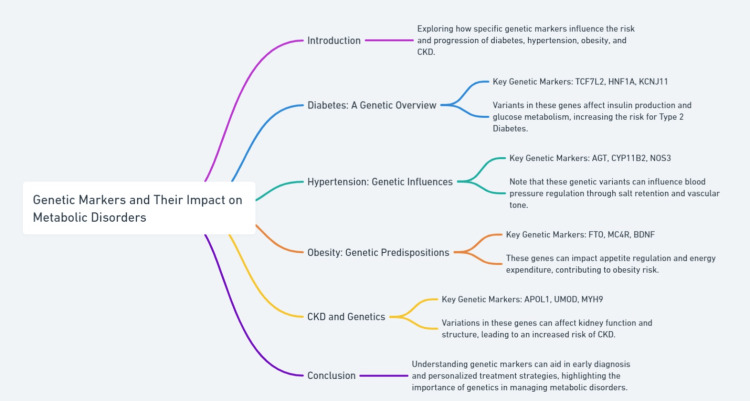
Exploring the impact of genetic markers on metabolic disorders. CKD: Chronic Kidney Disease TCF7L2: Transcription Factor 7 Like 2 HNF1A: Hepatocyte Nuclear Factor 1 Alpha KCNJ11: Potassium Voltage-Gated Channel Subfamily J Member 11 AGT: Angiotensinogen CYP11B2: Cytochrome P450 Family 11 Subfamily B Member 2 NOS3: Nitric Oxide Synthase 3 FTO: Fat Mass and Obesity-Associated Protein MC4R: Melanocortin 4 Receptor BDNF: Brain-Derived Neurotrophic Factor APOL1: Apolipoprotein L1 UMOD: Uromodulin MYH9: Myosin Heavy Chain 9 This image is generated by the authors.

Personalized medicine and pharmacogenomics hold promise for tailoring treatment strategies based on genetic profiles, potentially enhancing the efficacy and reducing the adverse effects of treatments for metabolic disorders. In diabetes, pharmacogenomic approaches are being explored to predict individual responses to anti-diabetic drugs, such as sulfonylureas and metformin, based on genetic variants influencing drug metabolism and action [29]. In obesity, genetic markers can guide the use of pharmacotherapy and bariatric surgery to achieve optimal weight loss outcomes [30]. For hypertension, identifying genetic variants that affect blood pressure response to antihypertensive drugs can improve treatment precision and control [31]. The integration of pharmacogenomics into clinical practice requires comprehensive genetic screening and robust predictive models but represents a frontier in achieving personalized healthcare for patients with metabolic disorders. A summary is given in Figure 2.

Advances in treatment and research

Expanding therapeutic options and technologies improving monitoring and self-management are advancing patient care [21].

Innovations in Pharmacotherapy

New anti-diabetic drug classes (SGLT2 inhibitors, GLP-1 agonists), improved insulin formulations, and novel treatments for obesity and kidney protection (e.g., bardoxolone) have expanded the pharmacopeia. 

Emerging Technologies in Monitoring and Management 

Continuous glucose monitoring, insulin pumps, and mobile health applications empower patients’ self-management. Wearables capturing physiologic signals and electronic health records enhance providers’ decision-making.

Future Directions in Research and Therapy

Key areas of investigation include expanding drug targets, stem cell therapies to regenerate insulin-producing beta cells in T1DM, microbiome-based interventions, and gene therapies for monogenic forms of obesity and diabetes [21].

Patient-centered care in managing chronic conditions 

Delivering patient-centered care, acknowledging psychosocial challenges, and aligning treatments with patient priorities are fundamental for improving outcomes in chronic disease management [32]. Ongoing education about disease processes, lifestyle behaviors, and skills for self-monitoring and medication management allows patients to participate actively in shared decision-making. 

Multidisciplinary Approach to Care 

Leveraging expertise across disciplines (physicians, nurses, dietitians, pharmacists, social workers, and mental health providers) connected by strong communication pathways better addresses the spectrum of biological and psychosocial needs.

Addressing Mental Health and Quality of Life

Screening for anxiety, and depression and assessing patient-reported outcomes provides insight into the impacts of disease burdens and treatments on emotional health and quality of life necessary to deliver comprehensive care.

Future directions and research gaps

Unmet Needs in Disease Management

Despite significant advancements in understanding and managing metabolic disorders, considerable unmet needs remain, particularly in achieving optimal disease control, preventing complications, and addressing the holistic needs of patients. For diabetes, there is a critical need for therapies that not only control blood glucose but also address the underlying beta-cell dysfunction and reduce cardiovascular risk without causing hypoglycemia [33]. In hypertension, challenges include improving adherence to medication and lifestyle interventions and developing strategies to manage resistant hypertension [16] effectively. Obesity management requires innovative approaches that sustain weight loss long-term and mitigate obesity-related comorbidities [34]. CKD management gaps include early detection, slowing progression, and addressing the high cardiovascular risk associated with CKD [35].

Promising Areas for Future Research

The exploration of novel therapeutic targets, such as gut microbiota modification, genetic and epigenetic interventions, and the harnessing of regenerative medicine and stem cell therapy, represents promising research avenues. In diabetes, the development of immunotherapies aiming to prevent or halt T1DM progression and the use of stem cells to regenerate beta-cell function offer exciting prospects [36]. The potential of precision nutrition to tailor dietary interventions based on genetic, metabolic, and microbiome profiles could revolutionize obesity and metabolic syndrome management [37]. For hypertension, investigating the role of the microbiome in blood pressure regulation and the development of targeted therapies based on genetic risk profiles are areas ripe for exploration [38]. CKD research is increasingly focusing on novel biomarkers for early detection and targeted therapies to prevent progression and manage complications [39].

Collaborative and Interdisciplinary Research Models

Addressing the complex challenges posed by metabolic disorders requires a collaborative and interdisciplinary approach, integrating expertise from genomics, bioinformatics, clinical medicine, public health, and behavioral sciences. Large-scale, multi-center clinical trials and longitudinal cohort studies that incorporate genetic, environmental, and lifestyle factors are essential for uncovering the multifactorial nature of these diseases. Additionally, the development of global consortia and partnerships between academia, industry, and government agencies can facilitate the translation of research findings into clinical and public health interventions. Such collaborative models can accelerate the discovery of novel diagnostics, therapeutics, and preventive strategies, ultimately leading to improved patient outcomes and health systems efficiency.

## Conclusions

This narrative review has illuminated the intricate interplay among diabetes, hypertension, obesity, and chronic kidney disease, emphasizing their collective impact on global health. The examination of their interconnected epidemiology, pathophysiology, and management strategies underscores the necessity for an integrated, multidisciplinary approach to treatment that encompasses not only medical intervention but also patient education, empowerment, and psychosocial support. As we advance, the promising horizons of personalized medicine, pharmacogenomics, and innovative research highlight the potential for significantly improved outcomes. However, addressing the substantial unmet needs in disease management and capitalizing on the synergies among these conditions require a concerted effort from healthcare professionals, researchers, and policymakers alike. By fostering collaborative research and adopting holistic care models, we can enhance the efficacy of interventions, ultimately reducing the burden of these metabolic disorders on individuals and societies worldwide.
